# Thioredoxin-interacting protein links endoplasmic reticulum stress to inflammatory brain injury and apoptosis after subarachnoid haemorrhage

**DOI:** 10.1186/s12974-017-0878-6

**Published:** 2017-05-11

**Authors:** Qing Zhao, Xudong Che, Hongxia Zhang, Pianpian Fan, Guanping Tan, Liu Liu, Dengzhi Jiang, Jun Zhao, Xiang Xiang, Yidan Liang, Xiaochuan Sun, Zhaohui He

**Affiliations:** 1 Department of Neurosurgery, the First Affiliated Hospital of Chongqing Medical University, 1 Friendship Road, 400016 Chongqing, China; 2 Department of Endocrinology, West China Hospital of Sichuan University, 37 Guo Xue Xiang, 610041 Chengdu, Sichuan China

**Keywords:** Subarachnoid haemorrhage, Early brain injury (EBI), Inflammation, Apoptosis, Thioredoxin-interacting protein, Endoplasmic reticulum stress

## Abstract

**Background:**

Early brain injury (EBI) is considered a major contributor to the high morbidity and mortality associated with subarachnoid haemorrhage (SAH). Both of sterile inflammation and apoptosis are considered the important causes of EBI. Recently, it was confirmed that thioredoxin-interacting protein (TXNIP) not only participates in inflammatory amplification but also stimulates the apoptosis signalling cascade pathway. However, whether the effects of TXNIP influence the pathogenesis of SAH remains unclear. Here, we hypothesize that TXNIP activity induced by endoplasmic reticulum stress (ER stress) may contribute to the pathogenesis of EBI through pro-inflammatory and pro-apoptotic mechanisms.

**Methods:**

A total of 299 male Sprague–Dawley rats were used to create SAH models. Resveratrol (RES, 60 mg/kg) and two TXNIP small interfering RNA (siRNA) were used to inhibit TXNIP expression. The specific inhibitors of ER stress sensors were used to disrupt the link between TXNIP and ER stress. SAH grade, neurological deficits, brain water content and blood–brain barrier (BBB) permeability were evaluated simultaneously as prognostic indicators. Fluorescent double-labelling was employed to detect the location of TXNIP in cerebral cells. Western blot and TUNEL were performed to study the mechanisms of TXNIP and EBI.

**Results:**

We found that TXNIP expression significantly increased after SAH, peaking at 48 h (0.48 ± 0.04, up to 3.2-fold) and decreasing at 72 h after surgery. This process was accompanied by the generation of inflammation-associated factors. TXNIP was expressed in the cytoplasm of neurons and was widely co-localized with TUNEL-positive cells in both the hippocampus and the cortex of SAH rats. We discovered for the first time that TXNIP was co-localized in neural immunocytes (microglia and astrocytes). After administration of RES, TXNIP siRNA and ER stress inhibitors, TXNIP expression was significantly reduced and the crosstalk between TXNIP and ER stress was disrupted; this was accompanied by a reduction in inflammatory and apoptotic factors, as well as attenuation of the prognostic indices.

**Conclusions:**

These results may represent the critical evidence to support the pro-inflammatory and pro-apoptotic effects of TXNIP after SAH. Our data suggest that TXNIP participates in EBI after SAH by mediating inflammation and apoptosis; these pathways may represent a potential therapeutic strategy for SAH treatment.

**Electronic supplementary material:**

The online version of this article (doi:10.1186/s12974-017-0878-6) contains supplementary material, which is available to authorized users.

## Background

Subarachnoid haemorrhage (SAH) is a serious neurological emergency associated with high morbidity and mortality [1]. Current studies show that early brain injury (EBI) is the overriding factor and that both neuroinflammation and apoptosis have been confirmed to contribute to EBI [2, 3]. EBI refers to the direct brain damage occurring within 72 h after SAH, including increased intracranial pressure, cerebral perfusion pressure disorder, blood–brain barrier (BBB) destruction and brain edema [2, 3].

Neuroinflammation is associated with various acute and chronic neurodegenerative diseases, including Alzheimer’s disease, Parkinson’s disease and SAH [3, 4]. Increased levels of cytokines have been found in the cerebrospinal fluid of SAH patients and are correlated with poor neurological outcomes [5]. Once bleeding occurs, neural immunocytes (microglia and astrocytes) rapidly react to the extravascular blood components that enter the cerebral parenchyma [6, 7]. The activated immunocytes then release various inflammation mediators such as Toll-like receptor 4 and interleukin-1β (IL-1β), thereby aggravating the secondary brain injury [8–10]. Various pro-apoptotic mechanisms are also reported to contribute to EBI after SAH and involve not only the activation of pro-apoptotic proteins but also the inhibition of anti-apoptotic factors [2, 3, 11]. Although some anti-inflammatory and anti-apoptotic strategies have been tested in preclinical and clinical trials, the mortality and disability burdens of SAH remain high, reminding us that the identification of appropriate and effective targets is still a major obstacle.

Thioredoxin-interacting protein (TXNIP), also known as thioredoxin-binding protein-2 or vitamin D_3_-upregulated protein 1, is a natural antagonist of thioredoxin (TRX) in vivo. Recent research has confirmed that TXNIP is the critical link between nod-like receptor protein 3 (NLRP3) inflammasome activation and inflammatory amplification [12]. Moreover, TXNIP can directly bind to and inhibit TRX function, leading to the activation of downstream apoptosis signalling pathways [13]. However, whether the effects of TXNIP influence the pathogenesis of SAH remains unclear. Previously, our work revealed that downstream factors in endoplasmic reticulum stress (ER stress) can induce neuronal apoptosis after SAH [14]. Intriguingly, recent studies have demonstrated that TXNIP expression can be significantly induced by ER stress at the transcriptional and post-transcriptional levels [15, 16]. The ER is the major site of protein folding, post-translational modification and assembly [17]. Although ER stress is primarily a self-protective signal transduction pathway, high-level ER stress could culminate in cell death via the activation of inflammation and apoptosis [18, 19]. Moreover, persistent and maladaptive ER stress is implicated in various human neurodegenerative diseases [20]. However, whether the induction of TXNIP by ER stress is involved in EBI after SAH remains unknown. Based on these findings, we utilized a TXNIP inhibitor, small interfering RNA (siRNA) and specific inhibitors of ER stress sensors to suppress TXNIP expression and ER stress–TXNIP signalling in order to assess the role of TXNIP in the progression of EBI after SAH.

## Methods

### Animals

**Fig. 1:**
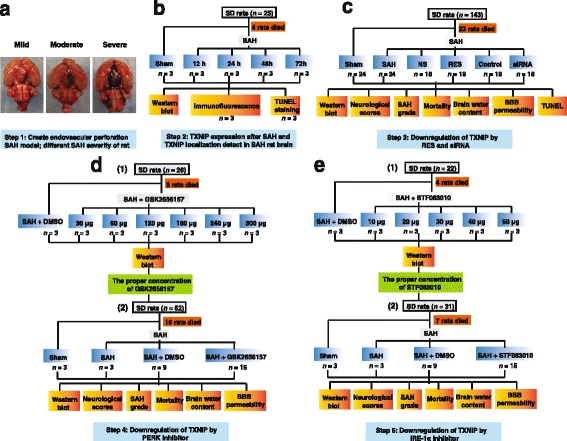
Endovascular perforation model of SAH and experimental design. **a** SAH severity levels; rats with a score below 5 points were excluded due to low SAH grade. **b**–**e** Experimental design of this study. *SD rats* Sprague–Dawley rats, *SAH* subarachnoid haemorrhage, *TXNIP* thioredoxin-interacting protein, *RES* resveratrol, *siRNA* small interfering RNA, *BBB* blood–brain barrier, *TUNEL* terminal deoxynucleotidyl transferase (TdT)-mediated dUTP nick end labelling, *PERK* protein kinase RNA-like ER kinase, *IRE1α* inositol-requiring enzyme-1α, *DMSO* dimethylsulfoxide

A total of 299 adult male Sprague–Dawley rats (280–350 g) were obtained from the Animal Center of Chongqing Medical University. Animals were maintained in a specific pathogen-free laboratory with regular 12/12 h light/dark cycles under controlled temperature and humidity conditions. The Animal Ethics and Use Committee of Chongqing Medical University approved all the experimental procedures. All surgical procedures were performed under deep anaesthesia to minimize suffering, in accordance with the recommendation guidelines in the Care and Use of Laboratory Animals of the National Institutes of Health. The experiments are designed and performed as in Fig. 1. Rats were divided into different groups as follows: sham (*n* = 33), SAH (*n* = 64), SAH + normal saline (NS, as a vehicle, *n* = 24), SAH + resveratrol (RES, *n* = 22), SAH + control siRNA (control, *n* = 23), SAH + TXNIP siRNA (*n* = 21), SAH + dimethylsulfoxide (DMSO, as a vehicle, *n* = 31), SAH + GSK2656157 (GSK, *n* = 42) and SAH + STF083010 (STF, *n* = 39).

### Endovascular perforation model of SAH

SAH animal models were created through endovascular perforation as described before [21]. Rats were anaesthetized with sodium pentobarbital (50 mg/kg) through intraperitoneal injection. Additional single doses (5 mg/kg) of pentobarbital were given to maintain anaesthesia when necessary. Sham-operated rats underwent identical procedures without the vessel puncture. After perforation, rats were kept in heated cages until recovery from anaesthesia. The electric heating pads were used to keep body temperature at 37 °C during and after the perforation.

### Resveratrol and TXNIP siRNA injection

Resveratrol (trans-3, 4′, 5-trihydroxystilbene, RES) was obtained from Sigma-Aldrich, St. Louis, MO (R5010, USA), and administered to the rats by intraperitoneal injection 1 h after puncture in a single dose of 60 mg/kg [22]. RES has been reported to significantly suppress TXNIP mRNA and protein expression and is capable of penetrating the BBB and reaching brain tissue rapidly [23, 24]. RES was dissolved in 50% ethanol and diluted with physiological saline (1 mL). Normal saline (1 mL) with 50% ethanol was used as the control.

Rats received TXNIP siRNA and control siRNA at 24 h before surgery via intracerebroventricular infusion as we described previously [14]. Two different TXNIP siRNA (Table 1) were designed as reported before [25, 26] and obtained from Guangzhou Ribo Biotechnology Co., Ltd. (Guangzhou, China). Briefly, 5 nmol of siRNA per rat in 6 μL sterile phosphate-buffered saline was inserted into the left lateral ventricles through a burr hole located at 1.5 mm posterior, 1.0 mm lateral and 3.2 mm under the bregma horizontal plane; the injection was performed with a sterile 10-μL Hamilton syringe and at a rate of 0.5 μL/min. Sham-treated and SAH animals also received a burr hole, but no siRNA injection was performed. After 10 min, the needle was removed and the burr hole was plugged carefully with bone wax.

**Table 1:** The sequences of two different TXNIP siRNA

Name	Sequences
siRNA 1 [25]	Sense: 5′-GCUGG AUAGACCUAAACAUTT-3′ Antisense: 5′-AUGUUUAGGUCUAUCCAGCTT-3′
siRNA 2 [26]	Sense: 5′-UGGUCACGUCGAAAUGAAUTT-3′ Antisense: 5′-TTACCAGUGCAGCUUUACUUA-3′
Control siRNA	Sense: 5′-UUCUCCGAACGUGUCACGUTT-3′ Antisense: 5′-ACGUGACACGUUCGGAGAATT-3′

### ER stress sensor inhibitor injection

Various stimuli could result in the activation of stress sensors in the ER transmembrane domain, including protein kinase RNA-like ER kinase (PERK) and inositol-requiring enzyme-1α (IRE1α) [27]. GSK2656157 (MedChem Express, HY-13820, China) is an adenosine triphosphate (ATP) competitive inhibitor that has been reported to effectively suppress PERK autophosphorylation via interaction with the PERK kinase domain [28]. However, the use of GSK2656157 in animal brain has not yet been reported before. Meanwhile, to avoid the BBB effect and to improve local drug concentration, SAH rats received GSK2656157 through intracerebroventricular injection at the same coordinate as TXNIP siRNA injection (1.5 mm posterior, 1.0 mm lateral and 3.2 mm below the horizontal plane of bregma). According to the previous study, GSK2656157 completely inhibits PERK autophosphorylation at 8 h after treatment [28]. Therefore, in this experiment, GSK2656157 was administered twice through two symmetrical bilateral burr holes, at 8 h before and after perforation. Ten microlitres of 1% DMSO in saline containing 30, 60, 120, 180, 240 or 300 μg of GSK2656157 was injected under anaesthesia at a rate of 0.5 μL/min by using a sterile 10-μL Hamilton syringe. Ten minutes later, the needle was removed and the burr hole was plugged with bone wax. The control group rats received 10 μL of 1% DMSO in saline without the inhibitor. The rats were quickly decapitated under deep anaesthesia at 24 h after surgery for Western blot.

STF083010, an IRE1α-specific inhibitor and cell-permeable molecule, was obtained from Sigma-Aldrich, St. Louis, MO (SML0409, USA). STF083010 has been reported to significantly inhibit IRE1α endoribonuclease activity, IRE1α-mediated X-box binding protein-1 (XBP1) mRNA splicing and TXNIP expression, without affecting IRE1α kinase in vitro and in vivo [15, 29]. A previous study found that the inhibition of IRE1α-mediated XBP1 splicing occurred at 6 h after STF083010 treatment [29]. STF083010 was also dissolved in DMSO at different concentrations: a total of 10 μL DMSO containing 10, 20, 30, 40 and 50 μg of STF083010 was administered twice a day, 6 h before and after perforation.

### Neurological scores, SAH grade and mortality assessment

Neurological scores were evaluated at 24 and 72 h in a blinded fashion using previously reported scoring methods [30]. An 18-point scoring system consisting of six subsets (spontaneous activity, spontaneous movements of four limbs, forelimb outstretching, wire cage wall climb, trunk touch reaction and vibrissae touch response) was used. After neurologic evaluation, the rats were sacrificed to quantify the severity of SAH according to a previously described grading scale [30]. Animals with a score below 5 points were excluded due to low SAH grade. Mortality was recorded after the operation.

### Blood–brain barrier (BBB) permeability and brain water content detection

BBB permeability assessment was based on the Evans blue extravasation [31]. At 72 h after perforation, Evans blue dye 4% (2.5 ml/kg) was injected into the rats’ left femoral vein under deep anaesthesia and allowed to circulate for 60 min. Spectrophotometry was used to quantify Evans blue dye at an excitation wavelength of 620 nm, an emission wavelength of 680 nm and a bandwidth of 10 nm. For brain water content measure, the brains were quickly divided into different portions under deep anaesthesia, then weighed immediately (wet weight) and kept in the oven for 72 h at 105 °C (dry weight). The brain water content percentage was recorded as the equation: (wet weight to dry weight)/wet weight × 100% [14].

### Immunofluorescence

Animals were sacrificed at 24 h after perforation for immunofluorescence analysis in accordance with methods reported in our previous work [14]. Ten-micrometre-thick sections of SAH rat brains were incubated with the following primary antibodies: TXNIP (1:50, Abcam, Cambridge, MA), NEUN (1:100, Merck Millipore), GFAP (1:50, Cell Signalling Technology, CST) and IBA1 (1:25, Abcam) overnight at 4 °C. Appropriate fluorescent secondary antibodies (Abbkine, Redlands, CA, USA) were used. Fluorescence microscopy (FV1200, Olympus, Japan) was used to detect TXNIP localization in neurons, microglia and astrocytes. For negative controls, the same staining procedures were conducted without primary antibodies.

### TUNEL staining

Fluorescent Terminal deoxynucleotidyl transferase (TdT)-mediated dUTP nick end labelling (TUNEL, Roche Inc, Mannheim, Germany) was performed according to the manufacturer’s protocol and previously reported methods [21]. TUNEL staining was used to detect TXNIP localization within apoptotic neurons in the hippocampus and cortex of SAH rats using fluorescence microscopy (Olympus, Japan). TUNEL-positive cells in five different fields per animal in the ipsilateral basal cortex (left) were counted under Olympus microscopy at ×400 magnification in a blind manner. The number of TUNEL-positive cells per square millimetre predicted the severity of brain injury. Negative controls were performed using a labelling solution without TUNEL reagent.

### Western blot

Rats were quickly decapitated under deep anaesthesia, and the left hemispheres were isolated for Western blot as described before [32]. The polyvinylidene fluoride (PVDF) membranes were incubated with primary antibodies as follows: TXNIP (1:500; Abcam, Cambridge, Mass), TRX1 (1:2000; Abcam), NLRP3 (1:1000; Abcam), cleaved Caspase-1 (1:500; Abcam), cleaved IL-1β (1:500; Abcam), cleaved Caspase-3 (1:1000; CST, Danvers, MA), BCL-2 (1:1000; CST), sXBP1 (1:100; Santa Cruz), phosphorylated-PERK (p-PERK, 1:200; Santa Cruz), eukaryotic translation initiation factor-2α (eIF2α) (1:100; Santa Cruz), phosphorylated-eIF2α (p-eIF2α, 1:500; Abcam), carbohydrate response element-binding protein (ChREBP) (1:100; Santa Cruz), activating transcription factor 5 (ATF-5) (1:2000; Abcam) and β-actin (NeoBioscience Technology Co., Ltd, Beijing, China). Membranes were incubated with the appropriate horseradish peroxidase-conjugated secondary antibodies, visualized with the enhanced chemiluminescent reagent kit (ECL, Engreen Biosystem Co., Ltd. Beijing, China) and analysed using the Fusion system (Fusion fx 7 Spectra, Vilber, France). Results are expressed as a percentage of the values for β-actin.

### Statistical analysis

Statistical analysis was performed using SPSS17.0 software. Mortality data was analysed using Fisher’s exact test. All of the other data were expressed as the mean ± standard error of the mean, and comparisons between multiple groups were performed by one-way analysis of variance (ANOVA). The Dunnett *t* test was used for comparisons between the control group and treatment group, and Student–Newman–Keuls was used for comparisons between pairs of treatment groups receiving different interventions. *p* < 0.05 was considered to be statistically significant.

## Results

### TXNIP expression increased after SAH

**Fig. 2:**
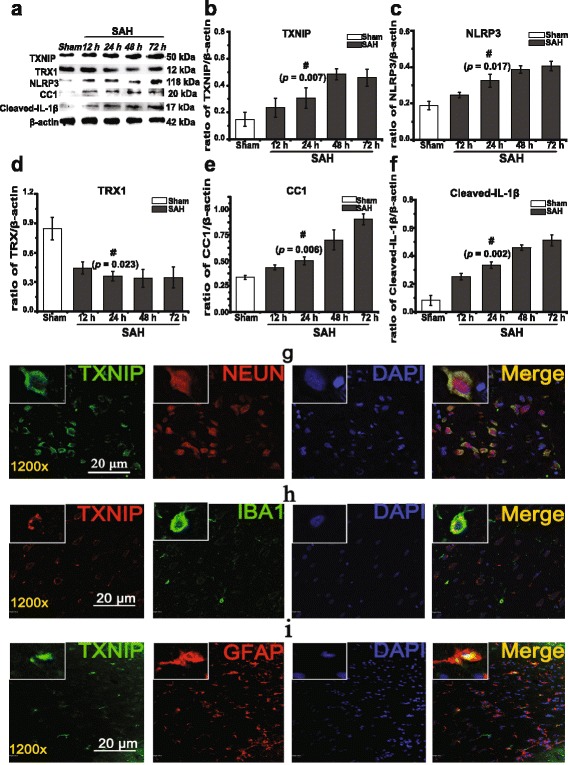
Expression of TXNIP and inflammatory factors at various time points after SAH; TXNIP expression in rat brain was assessed by histological fluorescence analysis. Representative Western blot of TXNIP, TRX1, NLRP3, cleaved Caspase-1 (CC1) and cleaved IL-1β (**a**). Densitometric quantification of protein band optical densities for TXNIP and associated proteins (**b**–**f**). TXNIP expression increased significantly after SAH (**a**, **b**). The expression of TXNIP, NLRP3, CC1 and IL-1β was also elevated after surgery and was accompanied by downregulation of TRX1 (**a, c**–**f**). Results were analysed using the Fusion system (fx 7 Spectra, Vilber, France) and expressed as a percentage of the values for β-actin. ^#^ *p* < 0.05 vs. sham. Representative histological immunofluorescence staining of TXNIP co-localized in neurons, microglia and astrocytes (**g**–**i**, detected by fluorescence microscopy, FV1200, Olympus, Japan). TXNIP was co-localized with NEUN-positive cells (neurons) (**g**, *n* = 3); TXNIP (*green*), NEUN (*red*), DAPI (nucleus, *blue*), original magnification ×1200. TXNIP was also co-localized with IBA1-positive cells (microglia) and GFAP-positive cells (astrocytes) in the subcortex (**h**, **i**, *n* = 3). **h** TXNIP (*red*), IBA1 (*green*), DAPI (*blue*), original magnification ×1200. **i** TXNIP (*green*), GFAP (*red*), DAPI (*blue*), original magnification ×1200. *Scale bars*: **g** 20 μm, **h** 20 μm, and **i** 20 μm. *TXNIP* thioredoxin-interacting protein, *TRX1* thioredoxin 1, *NLRP3 inflammasome* nod-like receptor protein 3 inflammasome, *IL-1β* interleukin-1β, *CC1* cleaved Caspase-1, *NEUN* neuron-specific nuclear protein, *DAPI* 4′,6-diamidino-2-phenylindole, *GFAP* glial fibrillary acidic protein, *IBA1* ionized calcium-binding adapter molecule 1

First, we created SAH models and performed Western blots to detect the expression of TXNIP and various inflammatory factors, including TXNIP, TRX1, NLRP3, cleaved Caspase-1 (CC1) and cleaved IL-1β, at different time intervals. Our results showed that TXNIP expression significantly increased after SAH compared with that of the sham group (0.15 ± 0.05), reached a peak at 48 h (0.48 ± 0.04, up to 3.2-fold, *p =* 0.007, Fig. 2a, b) and was still in a high level at 72 h (0.46 ± 0.06) after the surgery. Compared with the sham group, we also found that the expression of NLRP3 (*p =* 0.017), CC1 (*p =* 0.006) and cleaved IL-1β (*p =* 0.002) was elevated to varying degrees (Fig. 2a, c, e, f) and was accompanied by the downregulation of TRX1 (*p =* 0.023, Fig. 2d). These results indicated that the significantly elevated TXNIP and inflammatory cytokine expression were clearly induced by SAH.

### TXNIP is widely expressed in neurons and co-localizes with microglia and astrocytes after SAH

Immunofluorescence was used to detect TXNIP localization in SAH rat brains. We found that TXNIP was widely expressed in cytoplasm of neurons after SAH (*n* = 3, Fig. 2g). Both microglia and astrocytes play a crucial role in promoting neuroinflammation and secondary brain damage [33, 34]. However, there is no published literature to describe the histological localization of TXNIP in microglia and astrocytes after SAH. Therefore, we used specific markers of microglia and astrocytes to support these hypotheses. Importantly, we discovered for the first time that TXNIP was co-localized with microglia and astrocytes the cortex of SAH rats (*n* = 3, Fig. 2h, i).

### Downregulation of TXNIP by resveratrol (RES) and siRNA

**Fig. 3:**
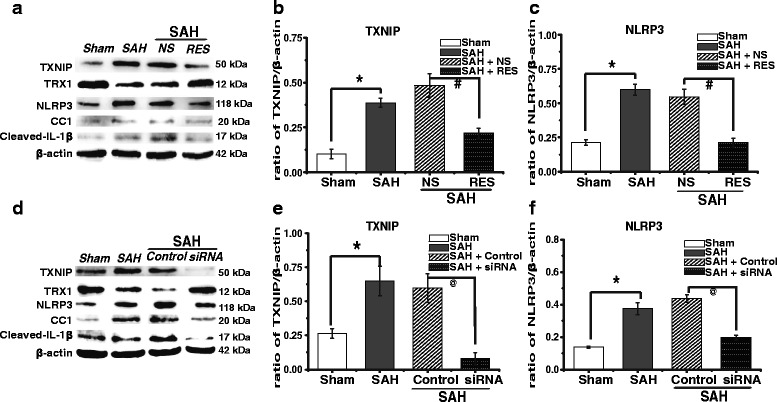
Expression of TXNIP and inflammatory factors after RES and TXNIP siRNA treatment. Representative Western blot of TXNIP, TRX1, NLRP3, cleaved Caspase-1 (CC1) and cleaved IL-1β (**a**, **d**). Densitometric quantification of protein band optical densities for TXNIP, TRX1, NLRP3, CC1 and cleaved IL-1β (**b**, **c, e**, **f**, and Additional file 1: Figure S1 and Additional file 2: Figure S2). The expression of TXNIP and inflammatory factors was reduced by RES and TXNIP siRNA treatment. Results were analysed using the Fusion system (fx 7 Spectra, Vilber, France). Results are expressed as a percentage of the values for β-actin. *p < 0.05 vs. sham; ^#^ *p <* 0.05 vs. SAH + NS; ^@^ *p <* 0.05 vs SAH + control siRNA. *TXNIP* thioredoxin-interacting protein, *TRX1* thioredoxin 1, *NLRP3 inflammasome* nod-like receptor protein 3 inflammasome, *IL-1β* interleukin-1β, *CC1* cleaved Caspase-1, *siRNA* small interfering RNA, *RES* resveratrol, *NS* normal saline

To provide further evidence of TXNIP’s pro-inflammatory role after SAH, both RES and TXNIP siRNA were used to suppress TXNIP expression. Our results showed that TXNIP blockage by RES decreased the generation of inflammatory factors (Fig. 3). These effects were associated with a reduction of TXNIP (*p* = 0.001), NLRP3 (*p* = 0.006), CC1 (*p* = 0.019) and cleaved IL-1β (*p* = 0.000) and improved TRX1 (*p* = 0.000) at 24 h after SAH (Fig. 3a–c and Additional file 1: Figure S1).

TXNIP is considered a promising therapeutic target. Although specific inhibitors and agonists of TXNIP would provide more convincing experimental evidences, such compounds are still lacking. Moreover, we cannot exclude the possibility that some other mechanisms may also mediate the observed protective effects. Previous studies have reported that RES may attenuate acute inflammatory brain injury after SAH by inhibiting other inflammatory mediator-dependent pathways [35]. We therefore used two reported siRNA mixtures to knock down TXNIP expression [25, 26]. We found that siRNA injection significantly reduced TXNIP expression (*p* = 0.003), as well as the expression of NLRP3 (*p* = 0.015), CC1 (*p* = 0.005) and cleaved IL-1β (*p* = 0.015), whereas TRX1 (*p* = 0.001) expression was enhanced (Fig. 3d–f and Additional file 2: Figure S2). Although we did not detect changes in TXNIP mRNA and immunofluorescence after RES and siRNA treatment, these results could indicate that TXNIP can aggravate EBI by triggering downstream inflammatory signalling pathways.

### TXNIP has a pro-apoptotic effect after SAH

**Fig. 4:**
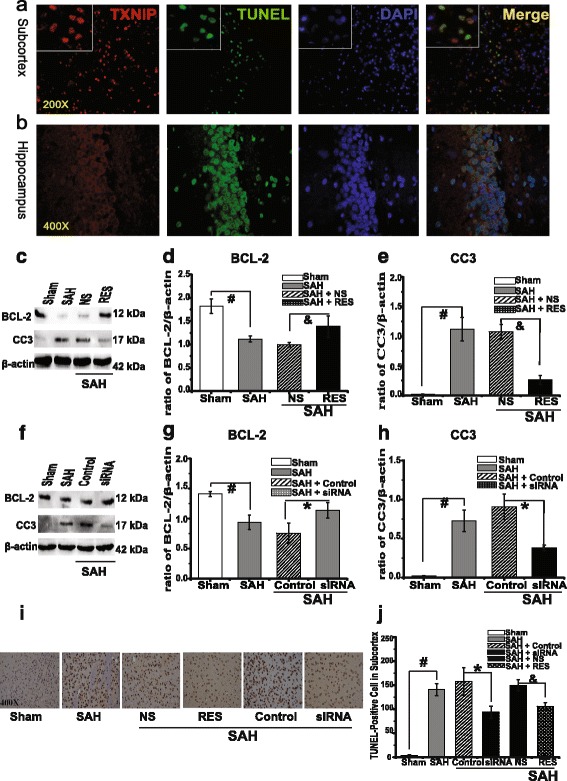
Histological fluorescence analysis of TXNIP, cleaved Caspase-3 (CC3) and BCL-2 expression after TXNIP siRNA and RES treatment. TXNIP was co-localized with TUNEL-positive cells in the hippocampus and subcortex (*n* = 3, IX71, Olympus, Japan); TXNIP (*red*), TUNEL (*green*), DAPI (nucleus, *blue*); original magnification: subcortex, ×200; hippocampus, ×400. *Scale bars*: 120 μm (**a**) and 60 μm (**b**). Representative Western blot of CC3 and BCL-2 (**c**, **f**). Densitometric quantification of protein band optical densities for CC3 and BCL-2 (**d**, **e**, **g**, **h**). RES and siRNA injection significantly inhibited CC3 expression and increased the generation of BCL-2. Results were analysed using the Fusion system (fx 7 Spectra, Vilber, France). Results are expressed as a percentage of the values for β-actin. Quantitative TUNEL-positive cells count after siRNA and RES treatment; original magnification ×400 (**i**, **j**, *n* = 5, Olympus microscopy, Japan). *Scale bars*: **i** 60 μm. ^#^ *p <* 0.05 vs. sham, *p < 0.05 vs SAH + control siRNA, &*p <* 0.05 vs SAH + NS. *TXNIP* thioredoxin-interacting protein, *RES* resveratrol, *siRNA* small interfering RNA, *CC3* cleaved Caspase-3, *BCL-2* B cell lymphoma (BCL)-2, *TUNEL* terminal deoxynucleotidyl transferase (TdT)-mediated dUTP nick end labelling, *DAPI* 4′,6-diamidino-2-phenylindole

A recent study reported that TXNIP exerts a pro-apoptotic effect by directly binding to TRX1, leading to the activation of apoptosis [13]. In order to confirm that TXNIP also has a pro-apoptotic after SAH, we labelled cells with TUNEL, a marker for cell death. TUNEL analysis revealed that TXNIP was widely co-localized with TUNEL-positive cells in both the hippocampus and the cortex (*n* = 3, Fig. 4a, b). Furthermore, TXNIP inhibition by RES and siRNAs treatment significantly downregulated cleaved Caspase-3 (CC3, *p* = 0.000 and *p* = 0.001) expression and elevated B cell lymphoma (BCL)-2 (BCL-2, *p* = 0.001 and *p* = 0.032) generation (Fig. 4c–h), while reducing the number of TUNEL-positive cells (Fig. 4i–j, *p <* 0.05). Although we did not detect the other apoptosis-related factors of TXNIP downstream signalling pathways and some researchers have previously reported that RES may reduce cell apoptosis through the activation of some anti-apoptotic pathways [22], these results indicated that TXNIP may aggravate apoptosis after SAH by triggering downstream apoptosis signalling pathways.

### Downregulation of TXNIP by PERK and IRE1α inhibition

Previous studies have confirmed that ER stress may be a principal response against neurodegeneration in various neurodegenerative diseases [20]. Our previous work also has found that the silencing of PERK downstream proteins could also attenuate EBI [14]. PERK, IRE1α and activating transcription factor-6 (ATF-6) are three important proteins in the ER transmembrane domain with multiple biological functions [27]. Recently, two research groups have found that irremediable ER stress could promote TXNIP expression through PERK at the transcriptional level and through IRE1α at the post-transcriptional level [15, 16]. However, ATF-6 has no effect on TXNIP expression [16]. Based on these findings, we speculated that TXNIP induced by ER stress may also occur after SAH. Therefore, we used a specific inhibitor of PERK and IRE1α to disrupt the links between TXNIP and ER stress.

**Fig. 5:**
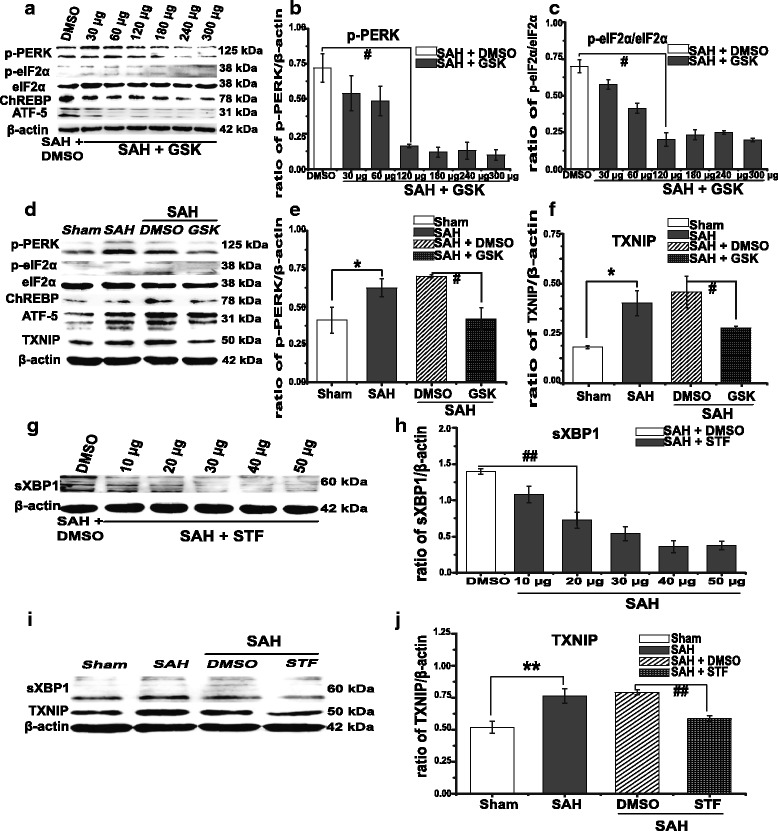
GSK2656157 treatment suppressed PERK autophosphorylation and TXNIP expression; STF083010 treatment inhibited IRE1α endonuclease activity and TXNIP expression. Representative Western blot and densitometric quantification of protein band optical densities for p-PERK, p-eIF2α/eIF2α, ATF-5, ChREBP, splicing XBP1 (sXBP1) and TXNIP (**a**–**j** and Additional file 3: Figure S3 and Additional file 4: Figure S4). TXNIP expression was downregulated by GSK2656157 and STF083010 treatment. Results were analysed using the Fusion system (fx 7 Spectra, Vilber, France). Results are expressed as a percentage of the values for β-actin. */**p < 0.05 vs. sham; ^#/##^ *p <* 0.05 vs. SAH + DMSO. *PERK* protein kinase RNA-like ER kinase, *IRE1α* inositol-requiring enzyme-1α, *DMSO* dimethylsulfoxide, *eIF-2α* eukaryotic translation initiation factor-2α, *ATF-5* activating transcription factor 5, *ChREBP* carbohydrate response element-binding protein, *XBP1* X-box binding protein-1, *TXNIP* thioredoxin-interacting protein

As we know, PERK is a type 1 transmembrane protein in the ER transmembrane domain that undergoes trans-autophosphorylation upon ER stress and further phosphorylates the eukaryotic translation initiation factor-2α (eIF-2α). Interestingly, recent studies have confirmed that TXNIP transcription is significantly regulated by the PERK–eIF2α pathway during ER stress; two signalling factors downstream of PERK–eIF2α, ATF-5 and ChREBP play an especially important role [15, 16]. In this study, we found that PERK phosphorylation was suppressed by GSK2656157 in a dose-dependent manner after SAH; the inhibition effect was apparent at a dose of 120 μg and pronounced at 180 μg to 300 μg (Fig. 5a, b, *p =* 0.000). The levels of eIF2α phosphorylation (*p* = 0.001), ChREBP (*p* = 0.000) and ATF-5 (*p* = 0.001) were also reduced (Fig. 5a, c and Additional file 3: Figure S3). We used 300 μg of GSK2656157 as the final dosage and found that TXNIP expression was significantly suppressed by GSK2656157 treatment (300 μg, *p* = 0.020, Fig. 5d–f and Additional file 4: Figure S4). Meanwhile, GSK2656157 treatment produced positive effect on protecting BBB permeability (Fig. 7c, *p <* 0.05). These results indirectly indicated that the protective effects of PERK inhibition were mediated by TXNIP downregulation, although we could not exclude the existence of other contributory mechanisms. Yan and colleagues recently found that PERK inhibition also exerts a neuroprotective effect through the activation of some protection signal factors [36]. That may explain why PERK inhibition showed no significant mortality decrease or improvement of neurological deficits in our work.

IRE1α is also a type 1 ER transmembrane protein with kinase and endoribonuclease activities that can induce XBP1 mRNA splicing through its endoribonuclease activity [37]. Interestingly, Lerner and colleagues recently reported that IRE1α can regulate TXNIP expression at the post-transcriptional level [15]. Thus, we speculated that IRE1α inhibition may also suppress TXNIP expression after SAH. STF083010 is an IRE1α-specific inhibitor that can completely inhibit IRE1α endoribonuclease-mediated XBP1 mRNA splicing [15, 29]. We found that STF-083010 inhibits IRE1α in a dose-dependent manner, at a minimal dose of 10 μg and with significant effect at 30 to 50 μg; the effect was reflected by XBP1 splicing (*p* = 0.000, Fig. 5g, h), an indirect measure, due to the difficulty of detecting IRE1α endoribonuclease activity [29]. Compared with the control group, animals treated with STF-083010 (50 μg) showed significantly inhibited TXNIP expression (*p* = 0.021) after SAH (Fig. 5i–j).

Above all, these results demonstrated that the inhibition of ER stress sensors may downregulate TXNIP expression after SAH. Therefore, these findings might indirectly suggest a potential link between ER stress, TXNIP and SAH.

### The analogue signalling pathway of TXNIP mediated by ER stress after SAH

The signalling pathway of TXNIP mediated by ER stress may be a novel therapeutic target in SAH-related EBI. ER stress occurs after SAH, activating PERK and IRE1α that are both ER stress sensors, as well as the downstream coherent factors. TXNIP expression is closely mediated by PERK and IRE1α. Firstly, ER stress activates the PERK-eIF2α pathway through activating the sensor PERK and its downstream transcription factors, ATF-5 and ChREBP, which significantly promote the transcription of TXNIP. In addition, hyperactivated IRE1α improves TXNIP mRNA stability through selective degradation of TXNIP-destabilizing microRNA-17 by its endoribonuclease activity. TXNIP is ubiquitously present in vivo and is a natural antagonist of TRX, while TRX plays an important role in redox homeostasis regulation [38]. Under various stresses, TXNIP can directly bind to TRX and disturb the redox homeostasis and TRX/apoptosis signal-regulating kinase-1 (ASK-1) inhibitory complex, leading to the reactive oxygen species (ROS) release and autophosphorylation of ASK-1 which is responsible for apoptosis. At the same time, TXNIP has been confirmed as a critical link for NLRP3 inflammasome activation, activating downstream Caspase-1 and IL-1β. Through the above mechanisms, TXNIP participates in EBI after SAH by mediating neuroinflammation and apoptosis.

### SAH severity and mortality

**Fig. 6:**
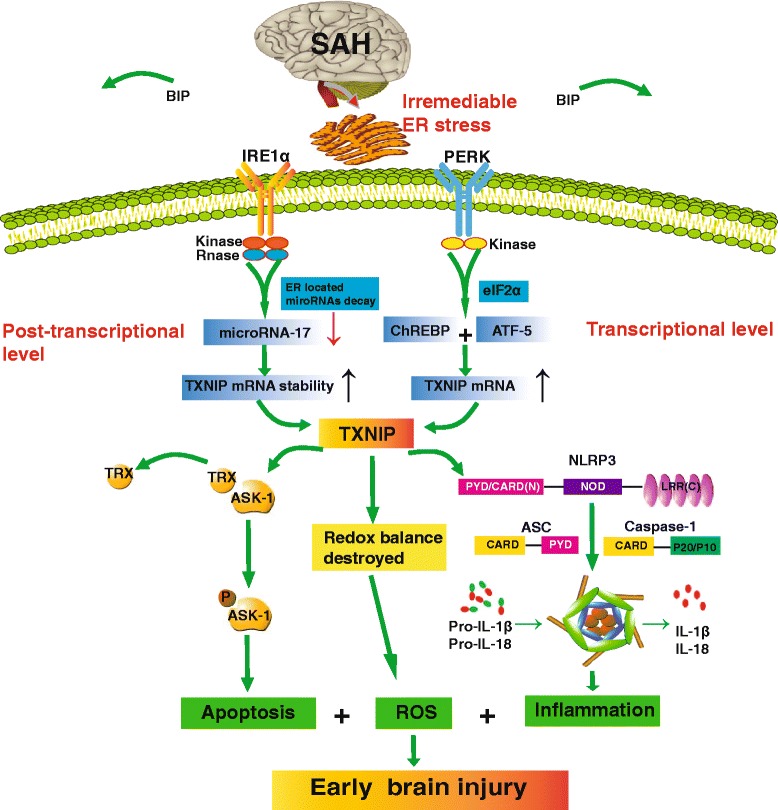
The analogue signalling pathway of TXNIP mediated by ER stress may be a novel therapeutic target in SAH-related EBI. *SAH* subarachnoid haemorrhage, *EBI* early brain injury, *ER stress* endoplasmic reticulum stress, *PERK* protein kinase RNA-like ER kinase, *IRE1α* inositol-requiring enzyme-1α, *eIF-2α* eukaryotic translation initiation factor-2α, *ATF-5* activating transcription factor 5, *ChREBP* carbohydrate response element-binding protein, *XBP1* X-box binding protein-1, *TRX* thioredoxin, *TXNIP* thioredoxin-interacting protein, *NLRP3 inflammasome* nod-like receptor protein 3 inflammasome, *IL-1β* interleukin-1β, *ASK-1* apoptosis signal-regulating kinase-1, *ROS* reactive oxygen species

Because SAH grade and mortality are prognostic indicators, they were recorded after surgery and treatment. SAH grading scores showed no significant difference between the surgery and treatment groups (Fig. 6a, *p =* 0.987), indicating that the extent of haemorrhage after SAH was similar to both control and treatment groups of rats. Mortality occurred mainly within 24 h after surgery in our study. A total of 22 rats died during or immediately after surgery due to severe SAH. Twenty rats were excluded because of their low SAH grade. No death was observed in the sham group. After surgery, 59 rats died within 24 h, with mortality rates which were calculated as follows: sham = 0% (0 of 33), SAH = 25.0% (16 of 64), NS = 25.0% (6 of 24), RES = 18.1% (4 of 22), control siRNA = 21.7% (5 of 23), TXNIP siRNA = 14.3% (3 of 21), DMSO = 22.6% (7 of 31), GSK2656157 = 21.4% (9 of 42) and STF083010 = 23.1% (9 of 39). However, there was no statistically significant difference in mortality between treatment groups (TXNIP siRNA vs. control siRNA, *p* = 0.701; RES vs. NS, *p* = 0.725; GSK2656157 vs. DMSO, *p* = 0.772; STF083010 vs. DMSO, *p* = 0.743) based on Fisher’s two-sided exact test.

### Neurological deficits

**Fig. 7:**
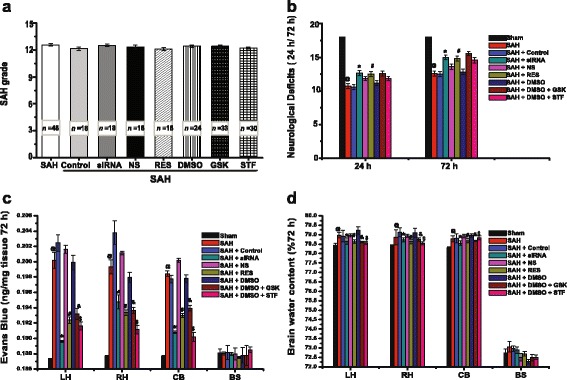
SAH grade and neurological deficits at 24 and 72 h; effect of TXNIP on SAH-induced BBB permeability and brain water content. SAH grading scores showed no statistically significant difference between the surgery and treatment groups (**a**, *n* = 6, each group). TXNIP siRNA and RES treatment increased the neurological score when compared with the control group, but GSK2656157 and STF083010 injection showed no significant score improvement (**b**). BBB disruption was also attenuated in three areas (LH, RH, CB) after treatment with TXNIP siRNA, RES or GSK2656157 (**c**). However, there were no significant differences between groups in the BS (**c**). The brain water content of LH, RH and CB was reduced by TXNIP siRNA, RES, GSK2656157 and STF083010 treatment (**d**, Additional file 5: Table S1). But, there were no statistical differences in BS between each control and treatment groups (**d**, Additional file 5: Table S1). ^*@*^ *p <* 0.05 vs. sham. *p < 0.05 vs. SAH+ control siRNA. ^#^ *p <* 0.05 vs. SAH + NS. &^/$^ *p <* 0.05 vs. SAH + DMSO. *SAH* subarachnoid haemorrhage, *RES* resveratrol, *siRNA* small interfering RNA, *NS* normal saline, *BBB* blood–brain barrier, *GSK* GSK2656157, *STF* STF083010, *DMSO* dimethylsulfoxide, *LH* left hemisphere, *RH* right hemisphere, *CB* cerebellum, *BS* brain stem

SAH significantly induced neurological dysfunction after surgery. Compared with the scores of the sham group (18.00 ± 0.00), the neurological scores of the SAH group were significantly lower at 24 and 72 h (24 h = 10.75 ± 0.37 and 72 h = 12.58 ± 0.36, *n* = 12, Fig. 7b, *p <* 0.05). However, there was no significant difference between groups (SAH, control siRNA, NS and DMSO groups, *p >* 0.05). Compared with the control group (24 h = 10.58 ± 0.36 and 72 h = 12.50 ± 0.38), neurological scores were improved after TXNIP siRNA treatment (24 h = 12.67 ± 0.40, *p* = 0.001 and 72 h = 15.00 ± 0.33, *p* = 0.000, Fig. 7b). The neurological scores of the RES treatment group (24 h = 12.50 ± 0.36, *p* = 0.016 and 72 h = 14.83 ± 0.39, *p* = 0.002) were higher than those of the control group (NS, 24 h = 11.16 ± 0.37 and 72 h = 12.83 ± 0.44, Fig. 7b). In contrast, there was no significant difference with GSK2656157 (24 h = 12.58 ± 0.36, *p* = 0.133 and 72 h = 15.58 ± 0.31, *p* = 0.068) or STF083010 (24 h = 11.91 ± 0.10 and 72 h = 14.42 ± 0.13, *p* = 0.160) compared with the control group (DMSO, 24 h = 11.18 ± 0.32 and 72 h = 14.58 ± 0.42, Fig. 7b). These results suggested that TXNIP inhibition could attenuate the neurological deficits of SAH rats, but PERK and IRE1α inhibition showed no significant improvement.

### BBB permeability

Evans blue dye extravasation was used to measure BBB permeability. SAH caused remarkable BBB disruption in the cerebral hemispheres, the cerebellum (CB) and the brain stem (BS) at 72 h post-surgery (Fig. 7c, *p <* 0.05). Compared with that observed in the control group, TXNIP siRNA injection significantly prevented BBB disruption, as reflected by the reduced levels of Evans blue dye in the left hemisphere (LH), right hemisphere (RH) and CB at 72 h (Fig. 7c, *p <* 0.05). RES treatment also attenuated BBB disruption in three areas (LH, RH and CB, Fig. 7c, *p <* 0.05). Both GSK2656157 and STF083010 had a protective effect on BBB permeability compared with the disruption observed in the DMSO control group (Fig. 7c, *p <* 0.05). However, there was no significant difference between groups in the BS (Fig. 7c, *p >* 0.05). These results showed that the inhibition of TXNIP, PERK and IRE1α could prevent BBB disruption in different regions after SAH.

### Brain water content

We found that SAH caused remarkably diffused brain swelling after surgery, especially at 72 h. Compared with that in the sham group, the brain water content was significantly worse in the LH, RH and CB at 72 h (Fig. 7d and Additional file 5: Table S1, *p <* 0.05). However, there is no significant difference observed in the BS between the control and treatment groups (Fig. 7d, *p >* 0.05). TXNIP siRNA injection significantly decreased the water content in the LH, RH and CB, when compared with rats in the control group (Fig. 7d and Additional file 5: Table S1, *p <* 0.05). SAH rats treated with RES showed a decrease in brain water content in the LH, RH and CB (Fig. 7d and Additional file 5: Table S1, *p <* 0.05). Brain water content was also significantly reduced by GSK2656157 and STF083010 treatment when compared with that of the control group (Fig. 7d and Additional file 5: Table S1, *p <* 0.05). These results demonstrated that TXNIP, PERK and IRE1α inhibition could reduce cerebral edema after SAH.

## Discussion

The present study proves that both pharmacological inhibition and gene knockdown of TXNIP significantly attenuated brain injury and improved early prognosis after SAH. We found that these effects were closely associated with TXNIP pro-inflammatory and pro-apoptotic signalling pathways. TXNIP was extensively co-localized with TUNEL-positive cells in both the rat hippocampus and subcortex after SAH. We also found for the first time that TXNIP is localized in microglia and astrocytes. These results represent critical evidence to support the pro-inflammatory and pro-apoptotic effects of TXNIP after SAH. PERK and IRE1α inhibition resulted in significant TXNIP suppression and attenuation of some prognostic indicators. More generally, we once again showed the important role of ER stress in EBI after SAH, highlighting the tight relationships between SAH, TXNIP and ER stress.

SAH results in severe neurological deficits, and there are few therapeutic drug targets available. Inflammation and apoptosis are regarded as the main causes in EBI after SAH. Although suppressing inflammation is generally neuroprotective in animal experiments, little success has been seen in clinical trials to date. Numerous apoptotic proteins are increased during this process, and many anti-apoptotic strategies have been developed for EBI treatment. Nevertheless, the mortality and disability of SAH remain unacceptably high, reminding us that there are some unknown mechanisms involved. Several studies recently reported that TXNIP plays dual pro-inflammatory and pro-apoptotic roles in ischaemic diseases [23, 39], but the relationship between TXNIP and SAH has not been elucidated. Kaya and colleagues reported that SAH induced TXNIP mRNA expression [40]. Therefore, we speculated that TXNIP may also participate in the pathogenesis of EBI. We found that TXNIP protein levels were significantly elevated and expressed in microglia and astrocytes during the initial phases of our SAH experiments. As described above, the neural immunocytes (microglia and astrocytes) play a major role in promoting neuroinflammation and secondary brain damage [33, 34]. Excessive microglial activation can aggravate cerebral haemorrhage-induced brain injury by inflammatory amplification [41, 42]. Inflammatory mediators could further activate astrocytes to induce secondary inflammatory responses [43]. In combination with that NLRP3 inflammasome is reported to be expressed in microglia cells and participates in the inflammatory brain injury of SAH [44, 45], our results provide a supplementary histological evidence to support that TXNIP may play a possible effect on EBI after SAH through NLRP3 inflammasome activation and inflammatory amplification.

Recent studies have found that TXNIP can interact closely with the leucine-rich repeats (LRRs) of the NLRP3 inflammasome, further inducing Caspase-1 and IL-1β activation under stress conditions and eventually promoting inflammatory amplification [12]. The NLRP3 inflammasome, as the best characterized inflammasome, consists of an NLRP3 scaffold, apoptosis-associated speck-like protein containing a CARD (ASC) and pro-Caspase-1 [46]. Under stress conditions, NLRP3 recruits ASC and pro-Caspase-1, further causing Caspase-1 autocatalytic activation and the downstream transformation of pro-IL-1β and pro-IL-18 into their cleaved and secreted forms [46]. In addition, TXNIP can directly bind to the active cysteine residue of TRX and further disturb the TRX/ASK-1 inhibitory complex, leading to ASK-1 release and pro-apoptotic effects [47]. TRX1 is a TRX protein that interacts closely with the N-terminal portion of ASK-1 and inhibits ASK-1-dependent apoptosis [48]. Researchers have found that TRX inhibition can induce neuronal apoptosis after cerebral ischaemia [38]. Likewise, we found that TRX1 expression was inhibited after SAH, which may be due to the co-localization of TXNIP with TRX1 and inhibition of TRX1 transcription [38]. We also found that TXNIP was widely expressed in SAH rat brain neurons. More importantly, TUNEL staining revealed that TXNIP was widely co-localized with apoptotic cells in the hippocampus and cortex after SAH. Brain cell apoptosis was reduced, and prognostic indicators were attenuated with inhibition of TXNIP. These results could support our speculation that TXNIP participates in EBI after SAH.

Recently, Oslowski and colleagues found that PERK significantly promotes TXNIP expression via the ATF-5 and ChREBP transcription factors under ER stress [16]. It has also been shown that hyperactivated IRE1α improves TXNIP mRNA stability through selective degradation of TXNIP-destabilizing microRNA via IRE1α endoribonuclease activity, eventually promoting TXNIP expression at the post-transcriptional level. When ER stress reaches a certain threshold, IRE1α selectively degrades four pre-microRNAs, including four microRNAs (miR-17, miR-34a, miR-96 and miR-125b) [49]. Among these, miR-17 has been confirmed to function as a TXNIP mRNA-destabilizing microRNA [15]. There are also some other mechanisms involved in regulating TXNIP expression, such as the p38 MAPK and forkhead box O1 transcriptional factor (FOXO1) pathways [50]. Of course, more research is still needed to identify additional potential mechanisms of TXNIP and ER stress in SAH.

Meanwhile, there are also some limitations of this current study and were listed as follows: (1) firstly, we did not detect a dose-dependent change of the mRNA, protein and immunofluorescence levels of TXNIP and downstream-associated factors after treatment with TXNIP siRNA, RES, GSK2656157 and STF083010; (2) secondly, we only tested the PERK phosphorylation levels. And a more thorough method is to detect the total-PERK levels and then calculate the ratios between the phosphorylation of PERK and total PERK, which will provide more favourable evidence to support our results; (3) in addition, ER stress can also occur in other pro-inflammatory immune or supporting cell types of the neurovascular unit, such as oligodendrocytes, vascular smooth muscle and endothelial cells. It is also possible that ER stress might induce pro-inflammatory activation of other cell types, which then can mediate indirect activation of TXNIP in neuronal cells, microglia and astrocytes in a paracrine matter; and (4) our Western blot experiments showed that TXNIP expression was the most obvious at 48 h. However, the immunolocalization experiment in Fig. 2 and the follow-up Western blot study for TXNIP was not done at the same 48 h time point used. Due to these, we should choose 48 h as the best testing point, while, combined with our found clinical and experimental observations, clinical symptoms appear and are significant at 24 h in patients and animals after intracranial aneurysm bleeding and SAH. So at the beginning of the experiment design, we aimed to observe the immunofluorescence and protein detection at 24 h. At the same time, TXNIP expression at 24 h was also significant when compared with that in the sham group. Therefore, the results of immunofluorescence and protein detection at 24 h may also be powerful to support the conclusions. These are limitations of our study design, and more work needs to be done in the future to address them.

## Conclusions

Our findings demonstrate that TXNIP participates in EBI after SAH by mediating neuroinflammation and apoptosis. The inhibition of TXNIP induced by ER stress alleviated SAH-induced inflammatory amplification and cell apoptosis and resulted in attenuation of some prognostic indicators, suggesting that TXNIP inhibition may represent a potential therapeutic strategy for SAH treatment. We speculate that the crosstalk between ER stress, apoptosis, inflammation and SAH will play an important role in SAH treatment and that TXNIP may be the critical link in these relationships. Of course, there are many questions that need to be addressed in our future work.

## Additional files

Additional file 1: Figure S1. TXNIP and inflammatory factor expression after RES treatment. Representative Western blot of TXNIP, TRX1, NLRP3, cleaved Caspase-1 (CC1) and cleaved IL-1β (a). Densitometric quantification of protein band optical densities for TRX1, CC1 and cleaved IL-1β (b-d). TXNIP and inflammatory factor expression were reduced by RES treatment. Analysed by Fusion (fx 7 Spectra, Vilber, France). Results were expressed as a percentage of the values of β-actin. *p < 0.05 vs. sham; #*p <* 0.05 vs. SAH+ NS. (EPS 1277 kb)

Additional file 2: Figure S2. The TXNIP and inflammatory factor expression after TXNIP siRNA treatment. Representative Western blot of TXNIP, TRX1, NLRP3, cleaved Caspase-1 (CC1) and cleaved IL-1β (a). Densitometric quantification of protein band optical densities for TRX1, CC1 and cleaved IL-1β (b-d). TXNIP and inflammatory factor expression were reduced by TXNIP siRNA treatment. Analysed by Fusion (fx 7 Spectra, Vilber, France). Results were expressed as a percentage of the values of β-actin. *p < 0.05 vs. sham; @*p <* 0.05 vs SAH+ control siRNA. (EPS 1213 kb)

Additional file 3: Figure S3. GSK2656157 treatment suppressed PERK autophosphorylation in a dose-dependent manner (a-c). Representative Western blot of p-PERK, p-eIF2α/eIF2α, ATF-5 and ChREBP (a). Analysed by Fusion (Fusion fx 7 Spectra, Vilber, France). #*p < 0.05* vs. SAH+ DMSO. (EPS 2706 kb)

Additional file 4: Figure S4. GSK2656157 treatment suppressed TXNIP expression (a-d). Representative Western blot of p-PERK, TXNIP, p-eIF2α/eIF2α, ATF-5 and ChREBP (a). Analysed by Fusion (Fusion fx 7 Spectra, Vilber, France). *p < 0.05 vs. sham; #/*p < 0.05 vs. SAH + DMSO. (EPS 2220 kb)

Additional file 5: Table S1. The brain water content. (DOC 38 kb)

## Abbreviations

EBI: Early brain injury
SAH: Subarachnoid haemorrhage
TXNIP: Thioredoxin-interacting protein
ER stress: Endoplasmic reticulum stress
siRNA: Small interfering RNA
BBB: Blood–brain barrier
IL-1β: Interleukin-1β
TRX: Thioredoxin
NLRP3 inflammasome: Nod-like receptor protein 3 inflammasome
PERK: Protein kinase RNA-like ER kinase
IRE1α: Inositol-requiring enzyme-1α
ATF-6: Activating transcription factor-6
RES: Resveratrol
eIF2α: Eukaryotic translation initiation factor-2α
TUNEL: Terminal deoxynucleotidyl transferase (TdT)-mediated dUTP nick end labelling
ATF-5: Activating transcription factor 5
ChREBP: Carbohydrate response element-binding protein
ASK-1: Apoptosis signal-regulating kinase-1
XBP1: X-box binding protein-1
