# Supplementary material for: Thioredoxin-interacting protein links endoplasmic reticulum stress to inflammatory brain injury and apoptosis after subarachnoid haemorrhage
Source: J Neuroinflammation. 2017 May 11;14:104. doi: 10.1186/s12974-017-0878-6 (PMC5426069; doi:10.1186/s12974-017-0878-6)
Supplement: Supplementary file 5 — The brain water content. (DOC 38 kb) [file 12974_2017_878_MOESM5_ESM.doc]

**Supplement data of table**

| **Table S1 Brain water content** | | | | |
| --- | --- | --- | --- | --- |
| Groups | LH | RH | CB | BS |
| Sham | 78.45 *±* 0.09% | 78.41 *±* 0.06% | 78.31 *±* 0.05% | 72.74 *±* 0.20% |
| SAH | 78.98 *±* 0.15% | 78.87 *±* 0.40% | 78.78 *±* 0.17% | 73.06 *±* 0.28% |
| Control siRNA | 78.92 *±* 0.31% | 79.12 *±* 0.24% | 78.81 *±* 0.24% | 72.97 *±* 0.15% |
| TXNIP siRNA | 78.66 *±* 0.17% | 78.73 *±* 0.11% | 78.55 *±* 0.12% | 72.89 *±* 0.17% |
| Normal saline | 78.96*±* 0.08% | 78.94*±* 0.07% | 78.93*±* 0.10% | 72.50 *±* 0.20% |
| RES | 78.63 *±* 0.09% | 78.64 *±* 0.13% | 78.68 *±* 0.12% | 72.69 *±* 0.12% |
| DMSO | 79.23 *±* 0.20% | 79.11 *±* 0.24% | 78.96 *±* 0.09% | 72.28 *±* 0.12% |
| GSK2656157 | 78.61 *±* 0.12% | 78.75 *±* 0.17% | 78.67 *±* 0.08% | 72.48 *±* 0.17% |
| STF083010 | 78.56 *±* 0.07% | 78.54 *±* 0.07% | 78.83 *±* 0.10% | 72.51 *±* 0.21% |
